# Simple-to-use nomogram for predicting the risk of syphilis among MSM in Guangdong Province: results from a serial cross-sectional study

**DOI:** 10.1186/s12879-021-06912-z

**Published:** 2021-11-29

**Authors:** Peizhen Zhao, Ziying Yang, Baohui Li, Mingzhou Xiong, Ye Zhang, Jiyuan Zhou, Cheng Wang

**Affiliations:** 1 Department of Biostatistics, State Key Laboratory of Organ Failure Research, Ministry of Education, and Guangdong Provincial Key Laboratory of Tropical Disease Research, School of Public Health, Southern Medical University, Guangzhou, China; 2 Dermatology Hospital, Southern Medical University, Guangzhou, China; 3 Kirby Institute, New South Wales University, Sydney, Australia; 4 Guangdong-Hong Kong-Macao Joint Laboratory for Contaminants Exposure and Health, Guangzhou, China

**Keywords:** Men who have sex with men, Syphilis, Nomogram

## Abstract

**Background:**

The purpose of this study was to develop and validate a simple-to-use nomogram for the prediction of syphilis infection among men who have sex with men (MSM) in Guangdong Province.

**Methods:**

A serial cross-sectional data of 2184 MSM from 2017 to 2019 was used to develop and validate the nomogram risk assessment model. The eligible MSM were randomly assigned to the training and validation dataset. Factors included in the nomogram were determined by multivariate logistic regression analysis based on the training dataset. The receiver operating characteristic (ROC) curves was used to assess its predictive accuracy and discriminative ability.

**Results:**

A total of 2184 MSM were recruited in this study. The prevalence of syphilis was 18.1% (396/2184). Multivariate logistic analysis found that age, the main venue used to find sexual partners, condom use in the past 6 months, commercial sex in the past 6 months, infection with sexually transmitted diseases (STD) in the past year were associated with syphilis infection using the training dataset. All these factors were included in the nomogram model that was well calibrated. The C-index was 0.80 (95% CI 0.76–0.84) in the training dataset, and 0.79 (95% CI 0.75–0.84) in the validation dataset.

**Conclusions:**

A simple-to-use nomogram for predicting the risk of syphilis has been developed and validated among MSM in Guangdong Province. The proposed nomogram shows good assessment performance.

**Supplementary Information:**

The online version contains supplementary material available at 10.1186/s12879-021-06912-z.

## Background

Syphilis remains an urgent public health priority worldwide [1]. There are an estimated 6 million new cases of syphilis globally among people aged 15 to 49 years each year. MSM are disproportionately burdened with syphilis infection globally [2], especially in low- and middle-income countries (LMICs), including China [3]. The overall prevalence of syphilis was 7.1% [4] and the incidence was 9.6 cases per 100 persons in 2015 among Chinese MSM [5].

Given the high risk of syphilis transmission among MSM, identifying MSM who are at high risk of syphilis infection can help design tailored behavior interventions to prevent the spread of syphilis [6]. Many studies have investigated the risk factors of syphilis infection, and it was found that MSM who had unprotected anal sex, commercial sex, multiple sexual partners or used drugs were more likely to be infected with syphilis in China [7–10]. However, previous studies only focused on identifying the associated factors without quantification and validation for the risk factors and failed to predict the probability of syphilis infection. Prediction of the probability of syphilis can help identify MSM who are at high risk, thus undergoing further intervention, testing and treatment. Therefore, the aims of this study were to investigate factors predictive of syphilis infection, and to develop and validate a simple-to-use nomogram for the prediction of syphilis infection among MSM in Guangdong Province in China.

## Methods

### Study design and participants

A governmental sentinel surveillance network has been set up for regular syphilis surveillance among MSM in four cities (Foshan, Jiangmen, Shenzhen, and Yunfu) in Guangdong Province in Southern China from April to June annually since 2017. (Additional file 1). Guangdong Province has a high burden of syphilis infection and has been consistently ranked first on syphilis incidence for the last decade among general population in China [11].

A standardized sentinel survey protocol designed by the Guangdong Provincial Centre for Sexually Transmitted Diseases Control was sent to sentinel surveillance network sites for data and sample collection each year. The sentinel surveillance was conducted by local public STD hospitals in each city. Each sentinel surveillance hospital has set up the MSM outreach team consisting of nurses, clinical physicians and public health physician. These outreach teams have extensive experiences of MSM outreach service, which can provide syphilis testing, condom promotion, comprehensive sexual health education, and behavioral intervention to reduce risks. The participants were recruited through offline outreach services, including pub, disco, tearoom and club. As a rule of thumb and based on the recommendation of World Health Organization, a minimum sample size of 200 participants was required for each sentinel surveillance hospital [12, 13]. Eligibility criteria for sentinel surveillance included: born biologically as a male, more than 18 years old, and had anal sex with a man at least once in the past 6 months. Among the eligible subjects, those who have already participated in this survey in other cities during the same year were excluded through face-face interview. Since we collected mobile phone numbers to inform the participants of the results of the syphilis test, we also excluded participants with the same mobile phone number.

### Data collection

The data was collected using paper questionnaires in this research. The questionnaire items were determined through discussions with STD experts, MSM and local sentinel surveillance hospital staff. We also piloted the survey with 20 volunteer MSM to test questionnaire items. The pilot data were not included in the final analysis. After providing written informed consent, MSM who agreed to participate in the study were referred to a separate, quiet room to complete the questionnaire with the help of a trained investigative assistant. We collected the phone number of each participant for notification of syphilis testing result. Each participant was given a disturbance allowance of 30 Yuan (about 4.3 USD) as an incentive for participation.

### Measures

#### Social-demographic and behavioral variables

Socio-demographic characteristic variables included age, marital status, residency, ethnicity and educational level. Sexual behavioral variables included commercial anal sex with men as well as vaginal sex with women, condom use consistently with men or women in the last 6 months, and condom use in last commercial sex. Drug use in lifetime and STD infection history in the past year were also collected. Drug use was defined as the using of any of the following drugs in the past year, cannabis, heroin, cocaine, crack, ecstasy, amphetamines, poppers, ketamine and methamphetamine. STD infection history was defined as the diagnosis of any of the following diseases in the past year: syphilis, chlamydia, gonorrhea, herpes and HPV infection. Condom use consistently over the last 6 months was defined as consistently using condoms in this study.

#### Syphilis laboratory testing

Blood samples were collected from all eligible participants for syphilis testing by nucleic acid amplification tests. The rapid plasma regains (RPR) test (Lizhu Biotech Inc, Zhuhai, China) was used for syphilis screening and Treponema pallidum particle agglutination (TPPA, Rongsheng Biotech Inc, Shanghai, China) was used for syphilis test confirmation. The syphilis test kits were approved by China’s State Food and Drug Administration. All participants would receive the syphilis test results through mobile text message within one week.

### Statistical analyses

All the data in this study were double-entered using the Epidata 3.0 software (EpiData Association from Denmark). All the dataset was randomly assigned into the training and validation datasets at the ratio of 2:1 (seed = 163,407) [17]. Categorical data (socio-demographic characteristics, sexual behaviors and syphilis infection characteristics) were presented as the number and percentage of MSM. The Chi-square test was used to compare qualitative variables between syphilis-infected and uninfected groups.

Nomogram is a graphical calculating device, which can reduce statistical risk factors to a single numerical estimate tailored to the individual patient’s profile and can generate an individual probability of an event [14, 15]. Analysis steps were as follows: First, a multivariate binary logistic regression model with stepwise variable selection was used to fit the prediction model based on the variables with a *P*-value less than 0.1 in univariate analyses using the training dataset [16, 17]. Second, a nomogram was developed depending on the independent significant syphilis infection risk factors, which were found in multivariate binary logistic regression using the training dataset [16, 17]. Each independent risk factor was assigned a corresponding score according to its location on the nomogram. The final risk score was calculated by adding up the score of each risk factor. A risk probability curve also demonstrated the association between total risk scores and syphilis infection. Third, to evaluate the performance and discrimination of the nomogram, we created a receiver operating characteristic (ROC) curve based on the total risk scores that were calculated by the nomogram in training and validation dataset. The optimal cut-off point for the predictive probability was determined by the maximization of Youden's index. In addition, the calibration was performed to assess performance of the nomogram by plotting the calibration curve to compare the observed prevalence and the predicted probability [18]. We used 5000 bootstrap resamples for calibration curves. Furthermore, the sensitivity, specificity, the positive predictive value (PPV), and the negative predictive value (NPV) were also calculated based on the optimal cut-off point. All analyses were conducted on R version 3.6.3 software (The R Project for Statistical Computing).

## Results

### Socio-demographic characteristics

In total, 2184 participants were recruited in this survey. Of those participants, the majority were between 18 and 35 years old (64.4%, 1406/2184), were never married (63.7%, 1392/2184), were of Han ethnicity (98.5%, 2152/2184), and were local residents (65.3%, 1427/2184). About one-third had a college degree or higher (37.5%, 819/2184). (Table 1).

**Table 1:** Characteristics of MSM who were syphilis positive or negative in Southern China, 2017–2019 (*N* = 2184)

Characteristics	Total (*N* = 2184)	Positive (*N* = 396)	Negative (*N* = 1788)	*χ*^2^	*P*
Age				27.61	< 0.001
18–25	598 (27.4)	71 (88.1)	527 (11.9)		
26–35	808 (37.0)	147 (81.8)	661 (18.2)		
36–45	456 (20.9)	105 (77.0)	351 (23.0)		
> 45	322 (14.7)	73 (77.3)	249 (22.7)		
Legal marital status				2.40	0.122
Never married	1392 (63.7)	239 (82.8)	1153 (17.2)		
Ever married/engaged	792 (36.3)	157 (80.2)	635 (19.8)		
Highest educational attainment				4.53	0.104
Less than high school	528 (24.2)	104 (80.3)	424 (19.7)		
High school	837 (38.3)	162 (80.6)	675 (19.4)		
College and above	819 (37.5)	130 (84.1)	689 (15.9)		
Ethnicity				5.77	0.016
Han	2152 (98.5)	385 (82.1)	1767 (17.9)		
Non-Han	32 (1.5)	11 (65.6)	21 (34.4)		
Length of time residence in current location				5.71	0.017
0 ~ 6 months	145 (6.6)	37 (74.5)	108 (25.5)		
Over 6 months	2039 (93.4)	359 (82.4)	1680 (17.6)		
Main venue used to seek male sexual partners				318.77	< 0.001
Internet	1324 (60.6)	83 (93.7)	1241 (6.3)		
Non-internet	860 (39.4)	313 (63.6)	547 (36.4)		
Local residence				66.25	< 0.001
Yes	1427 (65.3)	189 (86.8)	1238 (13.2)		
No	757 (34.7)	207 (72.7)	550 (27.3)		
Consistent condom uses in the past 6 months				5.82	0.016
Yes	1485 (68.0)	249 (83.2)	1236 (16.8)		
No	699 (32.0)	147 (79.0)	552 (21.0)		
Had commercial sex with men in the past 6 months				43.20	< 0.001
Yes	218 (10.0)	75 (65.6)	143 (34.4)		
No	1966 (90.0)	321 (83.7)	1645 (16.3)		
Condom use in the last commercial sex				10.44	0.001
Yes	132 (60.6)	7 (94.7)	125 (5.3)		
No	86 (39.4)	68 (20.9)	18 (79.1)		
Had sex with women in the past 6 months				0.47	0.493
Yes	339 (15.5)	57 (83.2)	282 (16.8)		
No	1845 (84.5)	339 (81.6)	1506 (18.4)		
Consistent condom uses with women in past 6 months				5.15	0.023
Yes	228 (67.3)	31 (86.4)	197 (13.6)		
No	111 (32.7)	26 (76.6)	85 (23.4)		
Drug use				0.21	0.650
Yes	45 (2.1)	7 (84.4)	38 (15.6)		
No	2139 (97.9)	389 (81.8)	1750 (18.2)		
Had infected STD in the past year				56.64	< 0.001
Yes	184 (8.4)	71 (61.4)	113 (38.6)		
No	2000 (91.6)	325 (83.8)	1675 (16.3)		

### Sexual behaviors and syphilis prevalence

The majority used internet to seek male sexual partners (60.6%, 1324/2184), and consistently used condom in the past 6 months (68.0%, 1485/2184). About one-tenth had commercial sex with men (10.0%, 218/2184) and had sex with women in the last 6 months (15.5%, 339/2184). The overall prevalence of syphilis was 18.1% (396/2184). The prevalence of syphilis in 2017, 2018 and 2019 was 12.4% (77/620), 18.9% (127/613) and 21.5% (192/891), respectively.

### Characteristics of the training and validation datasets

Of all the MSM participants, 1456 MSM were randomly selected as the training dataset, and the remaining 728 were regarded as the validation dataset. The differences between the characteristics of the training dataset and the validation dataset were not statistically significant, suggesting that the randomization into training and validation subset worked very well (Table 2).

**Table 2:** Characteristics of MSM participants in training and validation datasets in Southern China, 2017–2019 (*N* = 2184)

Characteristics	Training dataset (*N* = 1456)	Validation dataset (*N* = 728)	*χ*^2^	*P*
Age			6.12	0.106
< = 25	395 (27.1)	203 (27.9)		
26–35	542 (37.2)	266 (36.5)		
36–45	320 (22.0)	136 (18.7)		
> 45	199 (13.7)	123 (16.9)		
Legal marital status			1.28	0.257
Never married	940 (64.6)	452 (62.1)		
Ever married/engaged	516 (35.4)	276 (37.9)		
Highest educational attainment			0.14	0.931
Less than high school	353 (24.2)	175 (24.0)		
High school	554 (38.0)	283 (38.9)		
College and above	549 (37.7)	270 (37.1)		
Ethnicity			0.40	0.529
Han	1433 (98.4)	719 (98.8)		
Non-Han	23 (1.6)	9 (1.2)		
Length of time residence in current location			1.48	0.224
0 ~ 6 months	90 (6.2)	55 (7.6)		
Over 6 months	1366 (93.8)	673 (92.4)		
Main venue used to seek male sexual partners			0.19	0.665
Internet	878 (60.3)	446 (61.3)		
Non-internet	578 (39.7)	282 (38.7)		
Local residence			0.26	0.611
Yes	946 (65.0)	481 (66.1)		
No	510 (35.0)	247 (33.9)		
Consistent condom uses with men in the past 6 months			0.15	0.697
Yes	986 (67.7)	499 (68.5)		
No	470 (32.3)	229 (31.5)		
Had commercial sex with men in the past 6 months			0.04	0.840
Yes	144 (9.9)	74 (10.2)		
No	1312 (90.1)	654 (89.8)		
Condom use in the last commercial sex			0.06	0.804
Yes	94 (6.5)	45 (6.2)		
No	1362 (93.5)	683 (93.8)		
Had sex with women in the past 6 months			0.02	0.900
Yes	227 (15.6)	112 (15.4)		
No	1229 (84.4)	616 (84.6)		
Consistent condom uses with women in the past 6 months			0.67	0.413
Yes	156 (68.7)	72 (64.3)		
No	71 (31.3)	40 (35.7)		
Drug use			2.55	0.110
Yes	35 (2.4)	10 (1.4)		
No	1421 (97.6)	718 (98.6)		
Had infected with STD in the past year			0.15	0.703
Yes	125 (8.6)	59 (8.1)		
No	1331 (91.4)	669 (91.9)		

### Independent influencing factors

Table 3 lists the independent risk factors for syphilis infection among Chinese MSM by the multivariate logistic regression model. Stepwise logistic regression was used to determine independent risk factors of syphilis prevalence based on the variables with a *P*-value less than 0.1 in univariate analyses. The multivariate analysis indicated that MSM who were older (over 45 years old vs. 18–25 years old) (odds ratio (OR) = 1.90, 95%CI:1.15–3.14), sought sexual partners through non-internet route (OR = 10.49, 95%CI:7.38–14.91), had not consistently used condom in the last 6 months (OR = 1.41, 95%CI:1.03–1.96), had commercial sex with men in the last 6 months (OR = 2.50, 95%CI:1.59–3.94) and had been infected with STD (OR = 3.70, 95%CI:3.18–8.19) in the past year were more likely to have syphilis infection. (Table 3).

**Table 3:** Factors associated with syphilis infection among MSM in Southern China in the training dataset, 2017–2019 (*N* = 1456)

Characteristics	Univariate analysis		Multivariate analysis	
	*OR* (95%CI)	*P*	*OR* (95%CI)	*P*
Age				
< = 25	*Ref*		*Ref*	
26–35	1.50 (1.04–2.18)	0.030	1.29 (0.81–1.86)	0.329
36–45	2.11 (1.43–3.13)	< 0.001	1.62 (1.04–2.52)	0.032
> 45	2.02 (1.29–3.15)	0.002	1.90 (1.15–3.14)	0.012
Legal marital status				
Never married	0.92 (0.70–1.21)	0.536	–	
Ever married/engaged	*Ref*		–	
Highest educational attainment				
Less than high school	1.32 (0.94–1.85)	0.112	–	
High school	1.11 (0.81–1.51)	0.520	–	
College and above	*Ref*		–	
Ethnicity				
Han	*Ref*			
Non-Han	2.42 (1.01–5.76)	0.046		
Length of time residence in current location				
0 ~ 6 months	1.58 (0.96–2.59)	0.070	–	
Over 6 months	*Ref*		–	
Main venue used to seek male sexual partners				
Internet	*Ref*		*Ref*	
Non-internet	8.22 (5.98–11.29)	< 0.001	10.49 (7.38–14.91)	< 0.001
Local residence				
Yes	*Ref*			
No	2.49 (1.90–3.26)	< 0.001		
Consistent condom uses with men in the past 6 months				
Yes	*Ref*		*Ref*	
No	1.41 (1.07–1.85)	0.015	1.41 (1.03–1.96)	0.032
Had commercial sex with men in the past 6 months				
Yes	2.50 (1.71–3.63)	< 0.001	2.50 (1.59–3.94)	< 0.001
No	*Ref*		*Ref*	
Condom use in the last commercial sex				
Yes	*Ref*		–	
No	2.93 (1.34–6.41)	0.002	–	
Had sex with women in the past 6 months				
Yes	0.72 (0.49–1.07)	0.109	–	
No	*Ref*		–	
Consistent condom uses with women in the past 6 months				
Yes	*Ref*		–	
No	1.77 (0.83–3.77)	0.139	–	
Drug use				
Yes	0.92 (0.38–2.24)	0.853	–	
No	*Ref*		–	
Had infected with STD in the past year				
Yes	3.70 (2.52–5.43)	< 0.001	5.10 (3.18–8.19)	< 0.001
No	*Ref*		*Ref*	

### Development of a nomogram

**Fig. 1:**
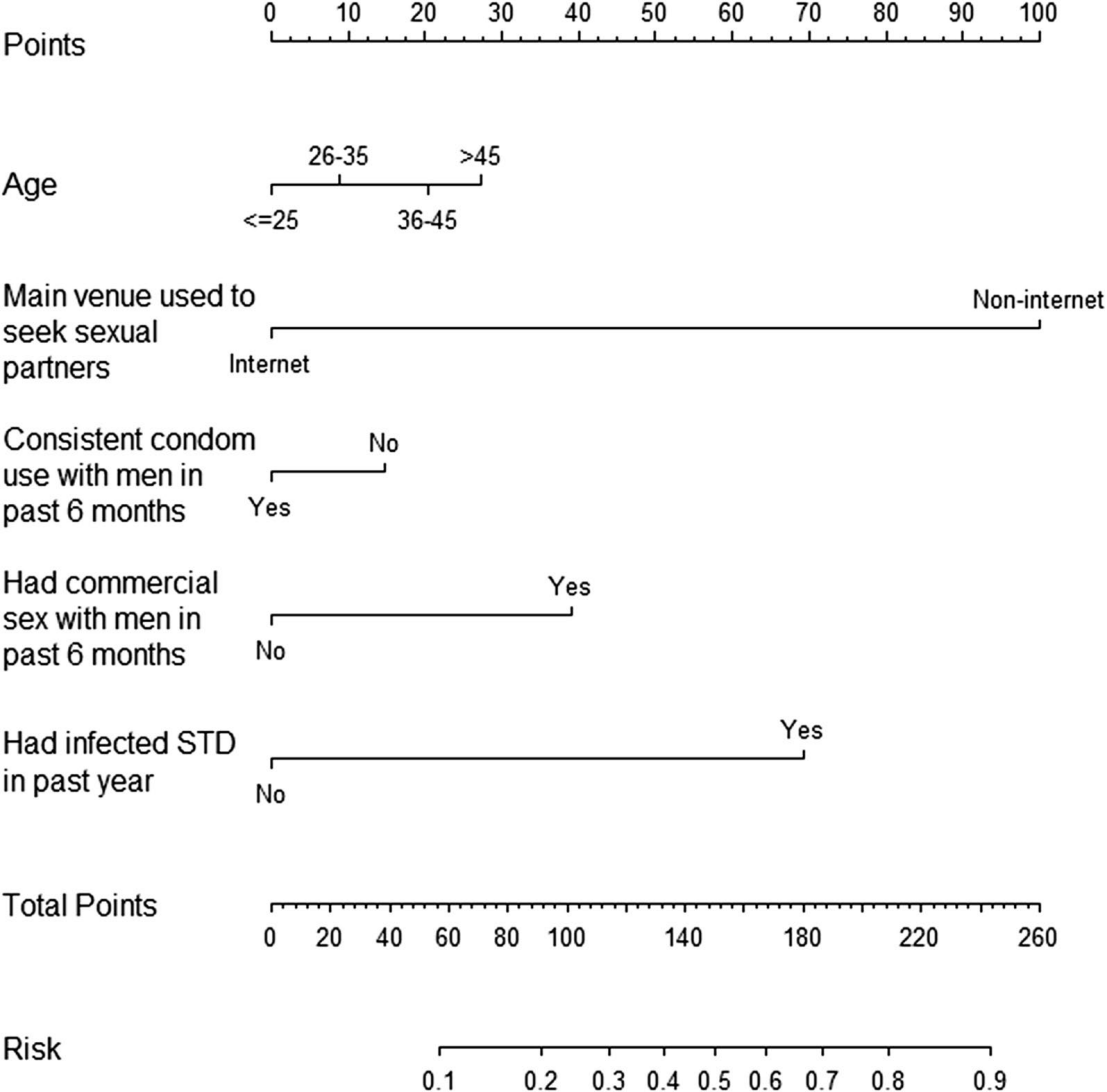
Nomogram for syphilis infection among MSM in Guangdong Province

On the basis of the multivariate logistic regression model, a predictive nomogram was constructed to evaluate the risk of syphilis. The scores for each factor of over 45 years old, seek sexual partners through non-internet route, inconsistent condom use with men in past 6 months, had commercial sex with men in past 6 months and had been infected with STD in past year were 27,100,15,39 and 69, respectively. (Fig. 1 and Additional file 1). A risk probability curve demonstrating the association between total risk scores and syphilis infection is shown in Additional file 1.

### Validation of the assessment accuracy of nomogram

**Fig. 2:**
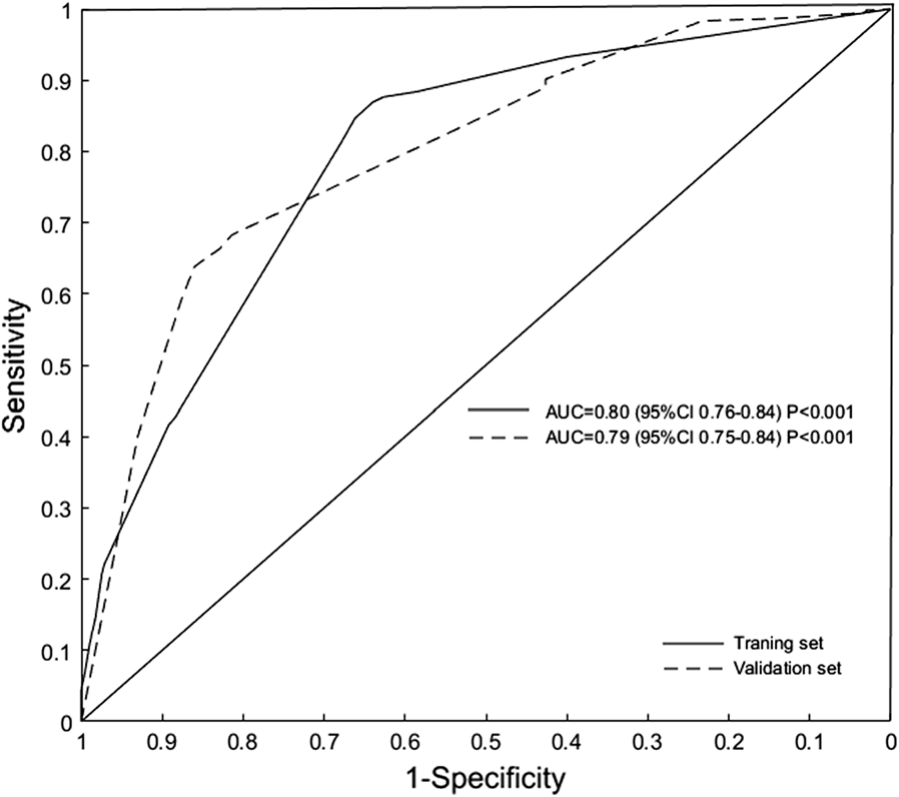
ROC curve validates the discriminatory power of the nomogram predictive of syphilis infection in training and validation dataset

**Fig. 3:**
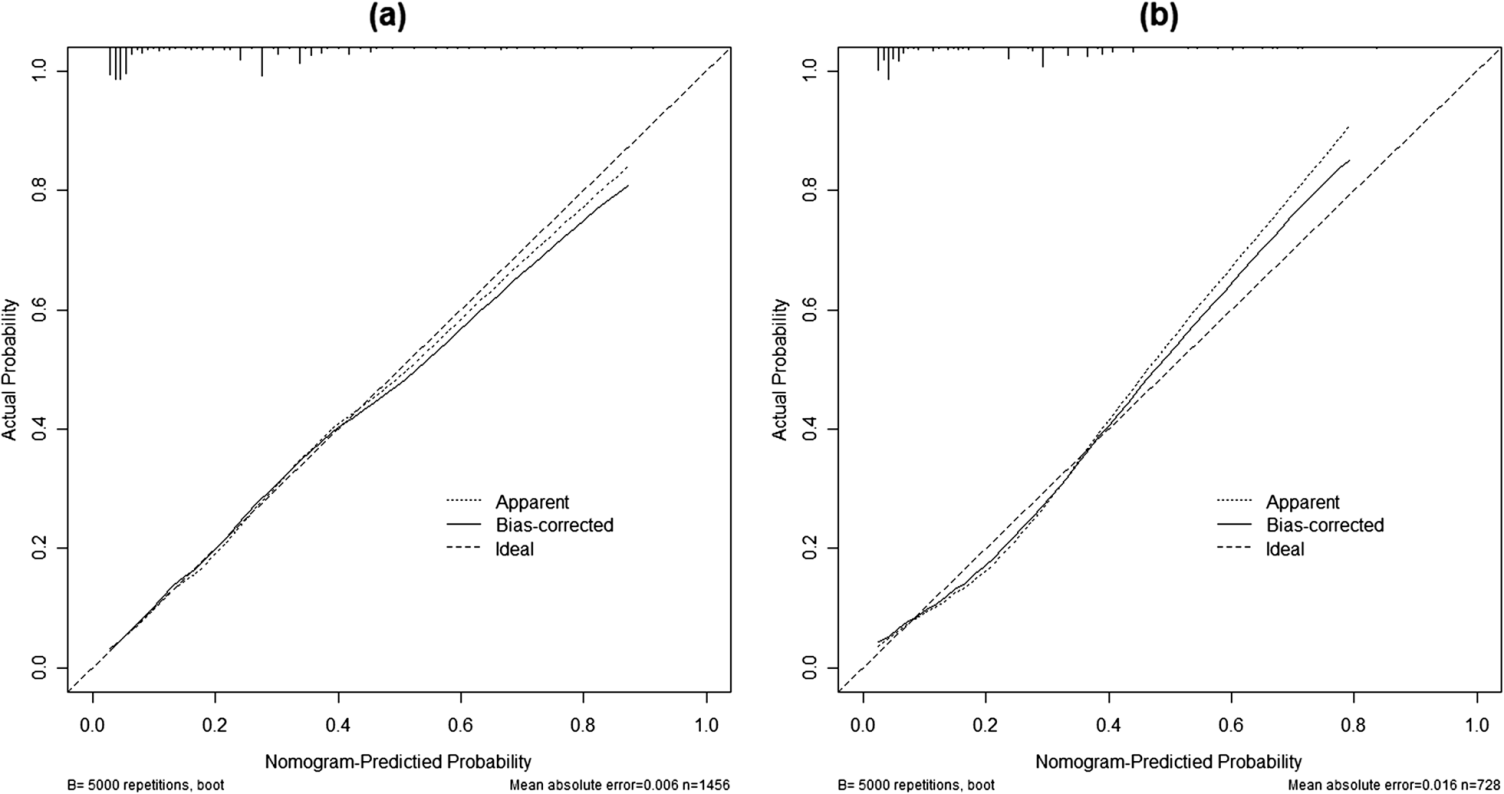
Calibration curve of the nomogram predictive of syphilis infection in MSM. **a** Calibration curve of the nomogram in training set. **b** Calibration curve of the nomogram in validation set

The C-index value was 0.80 (95% CI: 0.76–0.84) in the training dataset, indicating a fair power to identify syphilis infection (Fig. 2). As a cut-off point of 61.50 was determined by the maximization of Youden's index up to 0.509, it was shown that predictive accuracy of a nomogram comprised a sensitivity of 86.9%, a specificity of 64.0%, PPV of 35.2% and NPV of 95.6%. The C-index value was 0.79 (95% CI: 0.75–0.84) in the validation dataset (Fig. 2). The calibration curves of syphilis rates showed good agreement in training set and validation set, respectively (Fig. 3). In the validation set, we used the same cut-off value (61.5), the sensitivity, and the specificity, the PPV and the NPV were 81.4%, 68.3%, 94.5% and 35.6%, respectively.

## Discussion

In this study, a syphilis risk-prediction nomogram model was developed and validated using a relatively large MSM dataset in Guangdong Province. To the best of our knowledge, this is the first report of a nomogram incorporating significant risk factors for syphilis prediction among MSM globally. ROC indicated that this nomogram had significantly high sensitivity and specificity to distinguish individuals at a high risk of syphilis. This constructed nomogram model provides a simple-to-use and individualized tool for syphilis risk assessment, which can help target higher risk populations for timely syphilis testing and risk-reduction intervention among MSM.

In this study, we found that the overall prevalence of syphilis was 18.1%, and this prevalence among MSM was higher than the overall rate across China [4], and similar with that in developed cities among MSM in China [19]. The prevalence was higher than those in many developed countries, such as Argentina [20], Netherlands [21], Germany [22], and United Kingdom [23]. Our result also showed that the prevalence of syphilis was increasing year by year. The high prevalence may be due to the indulgence of unprotected anal or commercial sex intercourse in a large proportion of MSM in China [5]. The high prevalence of syphilis along with high-risk sexual behaviors may further accelerate the spread of the HIV and other STDs among MSM. Individual syphilis risk assessment can help identify populations at high risk for timely syphilis testing, treatment and risk-reducing behavior intervention.

In this study, five predictors were incorporated into the nomogram for the prediction of syphilis infection. We found that MSM who used condom consistently or had commercial sex in the past 6 months were more likely to be infected with syphilis [8–10, 19], which was consistent with other studies among Chinese MSM. Consistent and correct use of condoms has been proven to effectively reduce the risk of syphilis [24]. MSM who mainly seek male sexual partners through non-internet (traditional meeting places) were more likely to be infected with syphilis, this was consistent with other study [25]. This may be due to the fact that participants who seek sexual partners through non-internet route have more sexual partners and unprotected sexual behaviors [26]. Meanwhile, we also found that MSM who had STD history in the past year were more likely to be infected with syphilis, which was consistent with other study [27]. Unlike previous studies on syphilis infection prediction models, which only identified the associated risk factors, our study quantified and validated those risk factors, which can accurately predict the risk of syphilis. The discriminatory accuracy of our model appears to be good, which can be used for public health intervention, syphilis screening and personal syphilis risk assessment [26]. This nomogram risk evaluation model provides MSM with an accurate reflection of their vulnerability to syphilis infection due to their highly risky sexual behaviors. This tool can also be applied in other regions and countries with our method, while using their own MSM serial cross-sectional data to simulate their local parameters and develop a specialized syphilis risk assessment model.

The most important aspect of this syphilis nomogram model is its public health applicability and ease of use in a wide variety of health systems. As an example, a male aged 20 years who mainly used internet to seek sexual partners, consistently used condom with men in the last 6 months, had no commercial sex with men and ever had STD infection last year, would have a total risk score of 69 points, which corresponds to a probability of syphilis infection of 13%. In contrast, a male aged 40 years who mainly used non-internet methods to seek sexual partners, used condom inconsistently with men over the past 6 months, had commercial sex with men and ever had STD infection in the past year, would have a total risk score of 244 points, which corresponds to a probability of syphilis infection of 90%. The current findings support the risk assessment potential of the developed and validated nomogram, which is relatively straightforward to understand and can be obtained using a simple intake form in a time-saving way.

There were several limitations in the development of this syphilis infection nomogram model. First, there is a lack of several routinely available data in this study, such as the average number of sexual partners with men and women and the role of sexual behavior. Constructing a risk assessment model using both the factors identified in our model and other variables would thus be beneficial in creating a much more accurate syphilis risk prediction model. Second, the susceptibility of the self-reported behavioral data to social desirability bias might lead to misclassification in our study [28], especially those related to sexually risky behaviors. Third, the study was of cross-sectional design, thus there was generally no evidence of a temporal relationship between syphilis infection and factors. Last but not the least important, we only excluded the eligible subjects who have already participated in this survey in other cities through face-to-face interview and the same mobile phone number, there may still be repeated participants in the study.

## Conclusion

In conclusion, we developed and validated a simple-to-use nomogram for the prediction of syphilis among MSM. The nomogram model showed good assessment performance. The introduction of a syphilis risk nomogram model into syphilis control practice can help reduce the sexually risky behaviors and the prevalence of syphilis. Further studies to explore effective methods of promoting individualized syphilis risk assessments among MSM are warranted.

## Supplementary Information

**Additional file 1: Figure S1.** Map of Guangdong Province, China, highlighting the sentinel surveillance cities. **Figure S2.** Predicted probability curve. **Table S1.** Quantification of syphilis infection influencing factors.

## Data Availability

The datasets used and/or analyzed during the current study are available from the corresponding author on reasonable request.

## Abbreviations

MSM: Men who have sex with men
ROC: Receiver operating characteristic
STD: Sexually transmitted diseases
LMICs: Low- and middle-income countries
RPR: Rapid plasma regains
TPPA: Treponema pallidum particle agglutination
PPV: Positive predictive value
NPV: Negative predictive value
