# Supplementary material for: Simple-to-use nomogram for predicting the risk of syphilis among MSM in Guangdong Province: results from a serial cross-sectional study
Source: BMC Infect Dis. 2021 Nov 29;21:1199. doi: 10.1186/s12879-021-06912-z (PMC8628378; doi:10.1186/s12879-021-06912-z)
Supplement: Supplementary file 1 — Additional file 1: Figure S1. Map of Guangdong Province, China, highlighting the sentinel surveillance cities. Figure S2. Predicted probability curve. Table S1. Quantification of syphilis infection influencing factors. [file 12879_2021_6912_MOESM1_ESM.docx]

**Figure S1: Map of Guangdong Province, China, highlighting the sentinel surveillance cities**


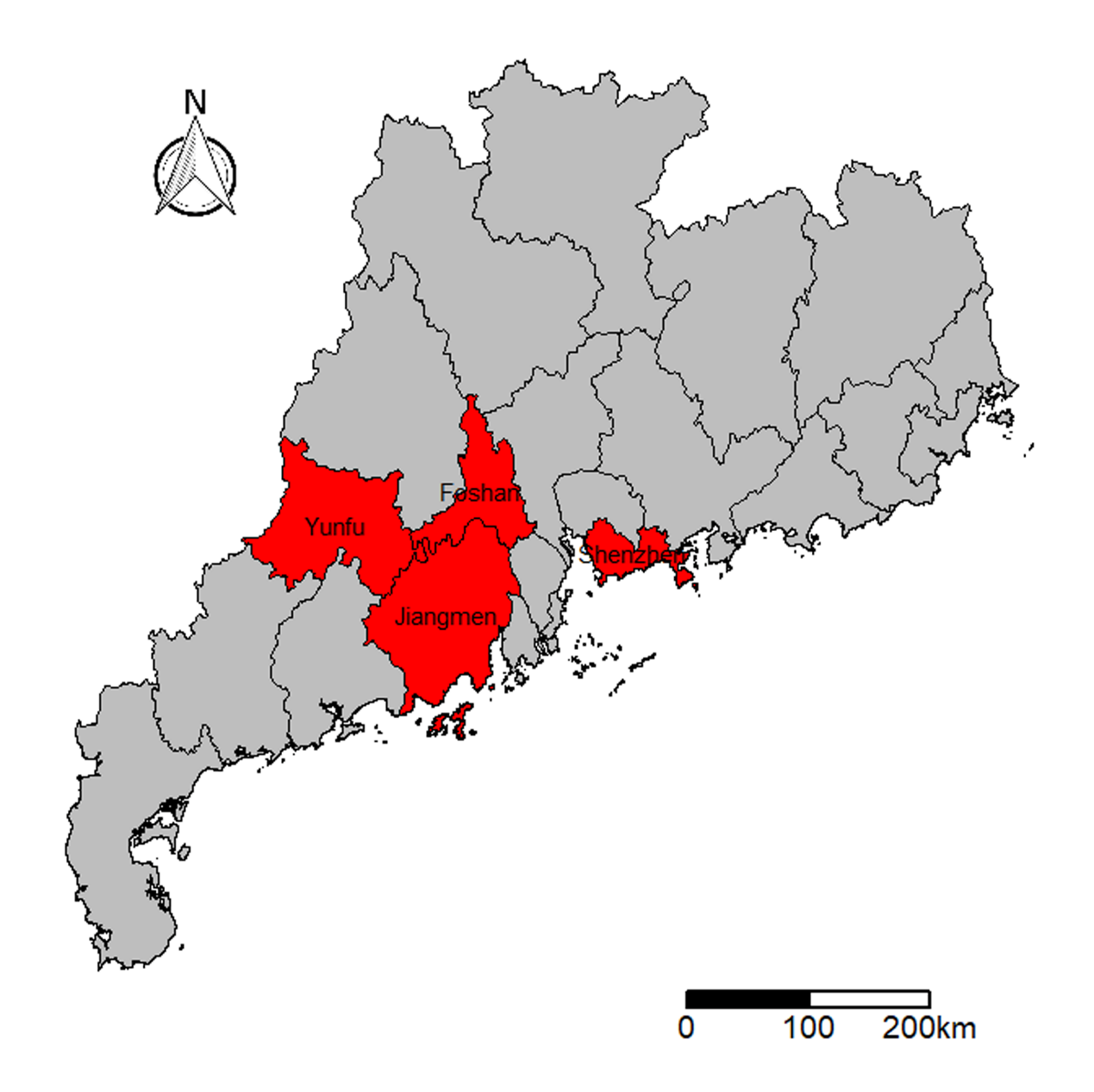


**Figure S2: Predicted probability curve**


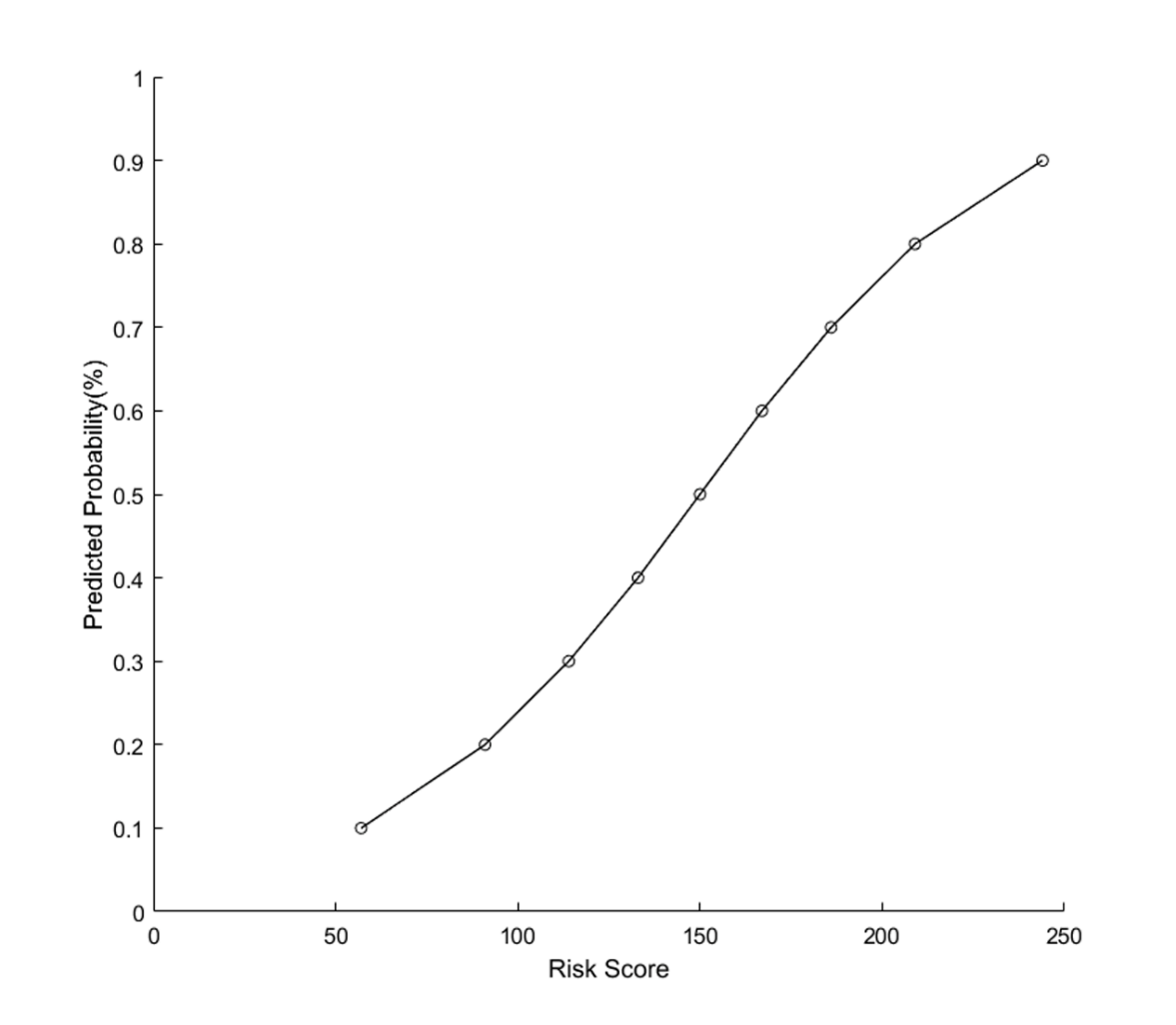


**Table S1** **Quantification of syphilis infection influencing factors**

| **Variables** | **Points** |
| --- | --- |
| **Age** |  |
| ≤25 | 0 |
| 26-35 | 9 |
| 36-45 | 21 |
| ＞45 | 27 |
| **Main venue used to seek sexual partners** |  |
| Internet | 0 |
| Non-internet | 100 |
| **Consistent condom use with men in past 6 months** |  |
| Yes | 0 |
| No | 15 |
| **Had commercial sex with men in past 6 months** |  |
| No | 0 |
| Yes | 39 |
| **Had infected STD in past year** |  |
| No | 0 |
| Yes | 69 |
